# Is BDNF sufficient for information transfer between microglia and dorsal horn neurons during the onset of central sensitization?

**DOI:** 10.1186/1744-8069-6-44

**Published:** 2010-07-23

**Authors:** James E Biggs, Van B Lu, Martin J Stebbing, Sridhar Balasubramanyan, Peter A Smith

**Affiliations:** 1 Department of Pharmacology and Centre for Neuroscience University of Alberta, Edmonton, Alberta, Canada; 2 Laboratory of MolecularPhysiology, NIH/NIAAA, Rockville, MD, USA; 3 School of Medical Sciences, RMIT University, Bundoora, Victoria, Australia; 4 Department of Pharmacology, University of Alberta, Edmonton, Alberta, Canada; 5 Department of Pharmacology and Centre for Neuroscience University of Alberta, Edmonton, Alberta, Canada

## Abstract

Peripheral nerve injury activates spinal microglia. This leads to enduring changes in the properties of dorsal horn neurons that initiate central sensitization and the onset of neuropathic pain. Although a variety of neuropeptides, cytokines, chemokines and neurotransmitters have been implicated at various points in this process, it is possible that much of the information transfer between activated microglia and neurons, at least in this context, may be explicable in terms of the actions of brain derived neurotrophic factor (BDNF). Microglial-derived BDNF mediates central sensitization in lamina I by attenuating inhibitory synaptic transmission. This involves an alteration in the chloride equilibrium potential as a result of down regulation of the potassium-chloride exporter, KCC2. In lamina II, BDNF duplicates many aspects of the effects of chronic constriction injury (CCI) of the sciatic nerve on excitatory transmission. It mediates an increase in synaptic drive to putative excitatory neurons whilst reducing that to inhibitory neurons. CCI produces a specific pattern of changes in excitatory synaptic transmission to tonic, delay, phasic, transient and irregular neurons. A very similar 'injury footprint' is seen following long-term exposure to BDNF. This review presents new information on the action of BDNF and CCI on lamina II neurons, including the similarity of their actions on the kinetics and distributions of subpopulations of miniature excitatory postsynaptic currents (mEPSC). These findings raise the possibility that BDNF functions as a final common path for a convergence of perturbations that culminate in the generation of neuropathic pain.

## 

In experimental animals, peripheral nerve damage, such as that generated by chronic constriction injury (CCI) of the sciatic nerve, induces pain-related behaviours that are accepted as a model for human neuropathic pain [1,2]. Seven or more days of CCI promotes release of cytokines, chemokines and neurotrophins at the site of nerve injury. These interact with first order primary afferent neurons to produce an enduring increase in their excitability [3-11]. The central terminals of these damaged afferents exhibit spontaneous activity and release additional cytokines, chemokines, neuropeptides, as well as ATP and brain derived neurotrophic factor (BDNF) [12-23] into the dorsal horn. These exert long term effects on dorsal horn excitability[14,24,25] and/or alter the state of activation of spinal microglial cells. Microglia stimulated in this way release of a further set of mediators, again including (BDNF) [13,14,17,18,24-30], that promote a slowly developing increase in excitability of second order sensory neurons in the dorsal horn of the spinal cord (Figure 1). This 'central sensitization' which develops progressively during CCI, [16,31-34] is responsible for the allodynia, hyperalgesia and causalgia that characterize human neuropathic pain [35]. Whereas microglial activation triggers pain onset, enduring activation of astrocytes is thought to be responsible for the maintenance of central sensitization [16,17,36-40]. Changes in thalamic and cortical physiology [35,41], long-term sensitization of peripheral nociceptors [16,35,42,43] and changes in descending inhibition from the rostral ventromedial medulla and periaqueductal grey [16,35,42,44-47] and are also involved. Although neuropathic pain can result from a variety of insults to peripheral nerves, including diabetic, postherpetic and HIV-AIDs related neuropathies [48,49], axotomy [3,4,50], nerve crush [51] or compression injury [52], the appearance of ectopic action potentials and spontaneous activity in primary afferent fibres seems to be the initial trigger that initiates central sensitization in many, if not all, types of peripherally generated neuropathic pain [35].

**Figure 1:**
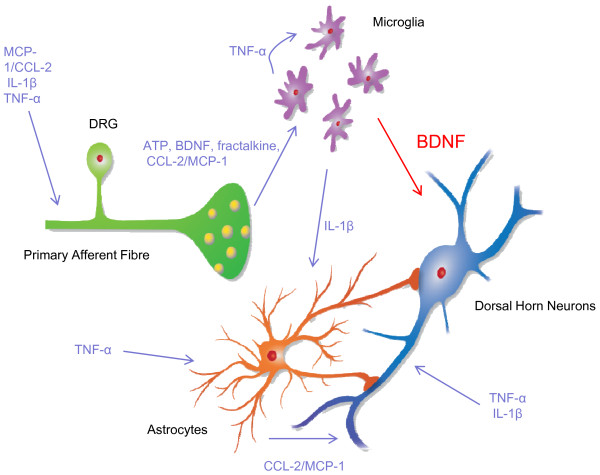
**Scheme to show interactions between primary afferents, dorsal horn neurons microglia and astroctyes in the context of chronic pain**. Literature citations supporting the illustrated interactions include; IL-1β, MCP-1/CCL-2 and TNF-α in acute and chronic excitation of primary afferents [3-9]; MCP-1/CCL-2, ATP, BDNF and fractalkine in microglial activation [13,18,59,60,64,65]; autocrine actions of TNF-α in microglia [120]; IL-1β release from microglia [55,57] and its actions on neurons [30,98]; BDNF release from microglia and its actions on neurons [14,21,24,25,61]; role of MCP-1/CCL-2 in astrocyte-neuron interactions [38], actions of TNF-α on astrocytes and neurons [30,58]. To the best of our knowledge actions of IL-1b on astrocytes in spinal cord has not been demonstated but there is evidence for this interaction in other neuronal systems [97,121].

Despite the documented importance of interleukin 1β (IL-1β) and tumor necrosis factor α (TNF-α) [8,29,30,53-58], MCP-1/CCL-2 [7,18,38], ATP [13,59], BDNF [14,21,24,25,60-62] and fractalkine [63-65] in central sensitization, findings to be reviewed below point to the possibility that BDNF is alone capable of bringing about one critical step; the interaction between activated microglia and neurons. It may therefore serve as a final common path for a convergence of perturbations that culminate in the generation of neuropathic pain [16,35] (Figure 1).

## A role for BDNF in CCI-induced increase in dorsal horn excitability

BDNF is increased in dorsal root ganglia (DRG) and spinal cord following crush or section of peripheral nerves [66-72]. It is released within the spinal cord following afferent fibre stimulation [23]. This release is Ca^2+ ^dependent and is favoured by high frequency burst activity [23,73]. Several lines of evidence are consistent with the central role for BDNF in the initiation of central sensitization [21,70,71,74,75]. For example, acutely applied BDNF sensitizes lamina II neurons to nociceptive input [76]. It also increases substance P release [77], enhances spinal responses to NMDA [78] and increases the frequency of miniature EPSCs (mEPSC) [79]. Intrathecal injection of BDNF produces hyperalgesia in normal mice whereas injection of antisense oligodeoxynucleotides directed against either BDNF or trkB, prevents inflammation-induced hyperalgesia [80]. Similarly, thermal hyperalgesia and allodynia produced by peripheral nerve ligation are attenuated in BDNF (+/-) heterozygous knock-out mice. They are also reduced following intrathecal injection of TrkB/Fc; a chimeric binding protein which sequesters BDNF [71].

The observations that 1) peripheral nerve injury attenuates GABAergic primary afferent depolarization [81], 2) that both CCI and BDNF reduce the amplitude of spontaneous and/or evoked IPSC's in dorsal horn neurons [14,33] and 3) that pharmacological blockade of the actions of inhibitory neurotransmitters promote allodynia [82-84] strongly implicate impediment of inhibitory neurotransmission in the development of central sensitization [16,81,84,85]. Mechanistically, this is thought to involve alterations in GABA release [33] as well as down regulation of the chloride transporter KCC2 in lamina 1 neurons by microglial-derived BDNF [14,86]. The resultant perturbation of the chloride gradient leads to attenuation of the inhibitory actions of GABA/glycine. In some neurons, the chloride gradient may actually reverse so that inhibition is converted to excitation [87]. Disinhibition also permits access of sensory information from low threshold Aβ fibres to pain projection neurons in lamina I [88-90]. This "opening of polysynaptic excitatory synaptic pathways" provides a physiological basis for the development of allodynia [16].

Although it has been reported that viral vector-driven expression of BDNF and grafting BDNF-expressing cells into the spinal cord reduces signs of pain associated with CCI [91,92] this may reflect analgesic actions within the midbrain [93-95]. Observations from our laboratory are consistent with a pro-nociceptive effect of BDNF, at least at the level of the spinal cord [24,25]. These and other observations raise the possibility that BDNF is alone capable of conveying many aspects of the communication between activated microglia and neurons during the onset of central sensitization. This appears to occur despite the presence and potential participation of mediators such as IL-1β [5,20,30,54,96-98], TNF-α [58,99], fractalkine [37,63,64,100,101], chemotaxic cytokine ligand 2 also known as monocyte chemoattractant protein 1 (CCL-2/MCP-1) [7,18,100,101]. These may exert their actions at other points in the central sensitization process (Figure 1) or perhaps function in a parallel fashion to BDNF in microglial - neuron interactions.

**Figure 2:**
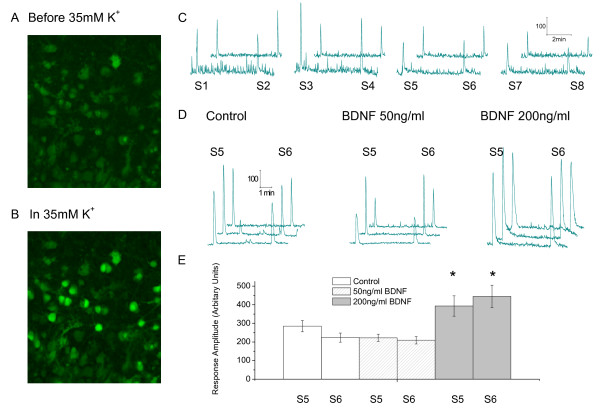
**Effect of electrical stimulation and 35 mM K^+ ^on intracellular Ca^2+ ^signals in *substantia gelatinosa *neurons**. **A**. Confocal image of fluo-4 filled neurons in *substantia gelatinosa *region of organotypic culture **B**. Second image of the same field as **A **after challenging with 35 mM K^+^. Note increased fluorescence intensity indicative of increases in intracellular Ca^2+^. **C**. Responses to a series of 5 sec 50 Hz stimuli (100 μS pulse width) to the dorsal root entry zone (S1 - S8) delivered at 5 minute intervals. Staggered traces show responses from two different neurons. Note despite the variability, responses S5 and S6 displayed fairly consistent amplitudes. These two responses were used for further comparisons. **D**. Typical S5 and S6 responses sampled from three typical neurons in control slices and in slices treated with 50 or 200 ng/ml BDNF. **E**. Summary of data from 32 neurons in control slices, 41 neurons from slices treated with 50 ng/ml BDNF and 22 neurons form slices treated with 200 ng/ml BDNF. Note enhanced responses in 200 ng/ml BDNF, * = *P < 0.05 *relative to appropriate control (One-way Anova with Tukey Kramer multiple comparisons test). Modified from reference [103]

BDNF immunoreactivity starts to increase 3 days after the initiation of peripheral nerve injury and its levels remain elevated for several weeks thereafter [66]. We therefore applied BDNF to spinal neurons for 5-6 days to test whether it produces a global increase in spinal cord excitability in a similar fashion to CCI [35]. This involved the use of an organotypic culture of rat spinal cord [102,103] that allowed us to expose mature neurons to BDNF for prolonged periods [24,25]. Effects on excitability were monitored by confocal Ca^2+ ^imaging using Fluo 4-AM. This was done in two ways, we either challenged neurons with high concentrations of extracellular K^+ ^(Figure 2A and 2B) or stimulated the dorsal root entry zone (50 Hz; 5 s; 100 μS pulse width) and observed the resultant elevation in intracellular Ca^2+^. Responses were collected from neurons (regions of interest) in control cultures or in cultures exposed to BDNF for 5-6 d. Ca^2+ ^responses were evoked by nerve stimulation once every 5 min and although there was variability in the amplitude of the responses, those evoked by the 5^th ^and 6^th ^stimulus (S5 and S6) were quite consistent (Figure 2C). Figure 2D compares sample S5 and S6 responses from control neurons with those from neurons cultured with 50 or 200 ng/ml BDNF for 6 days. Those evoked in the presence of the higher concentration of BDNF are clearly larger. The summary of responses of larger populations of neurons illustrated in Figure 2E shows that 200 ng/ml BDNF, but not 50 ng/ml, significantly increased the Ca^2+ ^responses (Figure 2E).

Using ELISA, we found that the ambient level of BDNF in control cultures was 26.2 ± 8.7 ng/ml (n = 3) [103]. Because this was not significantly changed after 6 d exposure of cultures to 50 ng/ml BDNF, where the measured BDNF concentration was 43.7 ± 7.3 ng/ml (n = 3; *P = 0.056*), this may explain the lack of effect of medium containing 50 ng/ml BDNF in Figure 2E. By contrast, the measured BDNF concentration was significantly increased to 92.4 ± 13.0 ng/ml (n = 3, *P < 0.002*) after 6 d exposure of cultures to medium containing 200 ng/ml BDNF. As mentioned, this concentration of BDNF promoted a significant increase in excitability was observed (Figure 2E). Metabolism, binding or breakdown of some of the exogenous BDNF by the cultures may explain the lack of correspondence between the applied and measured concentrations.

To test whether CCI would be expected to increase excitability in the cultures, we took advantage of the known role of microglia in central sensitization [12-20,104]. We found that the excitability of cultures was increased when they were exposed to medium conditioned by exposure to lipopolysaccharide activated microglia (activated microglia conditioned medium aMCM [105]). This increase in excitability could be prevented by sequestering BDNF with the binding protein TrkBd5 [25,106]. This implicates BDNF in the increased excitability produced by aMCM and supports its role as a mediator of pain centralization.

In another series of experiments, we noted that excitability of the cultures could also be increased by 6-8 d exposure to 100 pM interleukin 1β (IL-1β) [98]. Despite this, we do not believe that this cytokine plays a major role in the microglia - neuron interactions that lead to central sensitization. This is because the actions of CCI and BDNF display remarkable similarity at the cellular level [24,25] whereas the cellular actions of IL-1β are quite different from those seen with CCI [98] (see below).

## BDNF and increased excitability of superficial laminae

**Figure 3:**
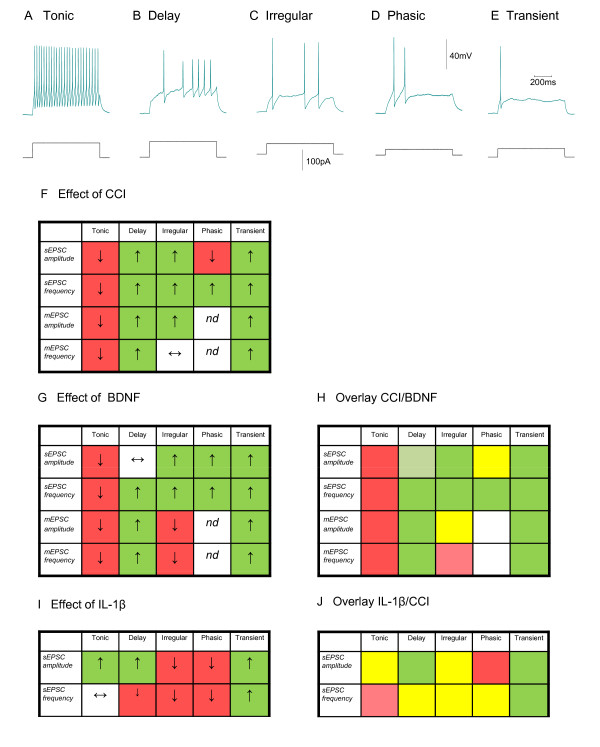
**Neuron types and injury footprints produced by CCI and BDNF**. **A-E **Firing patterns of tonic, delay, irregular, phasic and transient neurons in response to depolarizing current commands. **F**. Injury footprint produced by CCI. Neuron types are listed across the top of the scheme and four indices of excitatory synaptic transmission are listed to the left. Neuron specific parameters increased (↑; such as sEPSC amplitude in delay neurons) are coded green. Neuron specific parameters decreased (↓; such as sEPSC amplitude in tonic neurons) are coded red. Data from [107]. nd = not determined. **G**. Injury footprint produced by BDNF. Neuron types are listed across the top of the scheme and four indices of excitatory synaptic transmission are listed to the left. Neuron specific parameters increased (↑; such as sEPSC amplitude in delay neurons) are coded green. Neuron specific parameters decreased (↓; such as sEPSC amplitude in tonic neurons) are coded red. Data from [24]. **H**. Overlay of the injury footprints from **F **and **G**, similarities between the actions of CCI and BDNF treatment show up as clear green or red squares. Yellow squares illustrate the few parameters which appear to be altered in a different way by BDNF compared to CCI. 2 out 20 (10%) of squares are yellow. **I**. Injury footprint produced by IL-1β. Neuron types are listed across the top of the scheme and indices of excitatory synaptic transmission SEPSC amplitude and frequency are listed to the left. Neuron specific parameters increased (↑) are coded green. Neuron specific parameters decreased (↓) are coded red. **J**. Overlay of IL-1β injury footprint from **I **with sEPSC amplitude and frequency data from **A**. Yellow squares illustrate the parameters which appear to be altered in a different way by IL-1β compared to CCI. 5 out of 10 (50%) of squares are yellow. This analysis indicates that the consequences of CCI are mimicked more accurately by BDNF than by IL-1β.

We have found that CCI produces a specific set of changes in excitatory synaptic transmission in lamina II. Neurons in this region can be classified according to five electrophysiological phenotypes according to their firing pattern in response to depolarizing current. These are defined as tonic, delay, irregular, phasic and transient firing neurons (Figure 3A-E) [24,102,107,108]. Although CCI has minimal effects on the intrinsic membrane properties of these five neuron types, it produces a discrete pattern of changes in excitatory transmission across the whole population; the amplitude and frequency of both miniature and spontaneous excitatory postsynaptic currents (mESPC and sEPSC) are increased in most neuron types but are reduced in tonic firing neurons (Figure 3F). This pattern of changes may be referred to as an 'injury footprint' [107].

**Figure 4:**
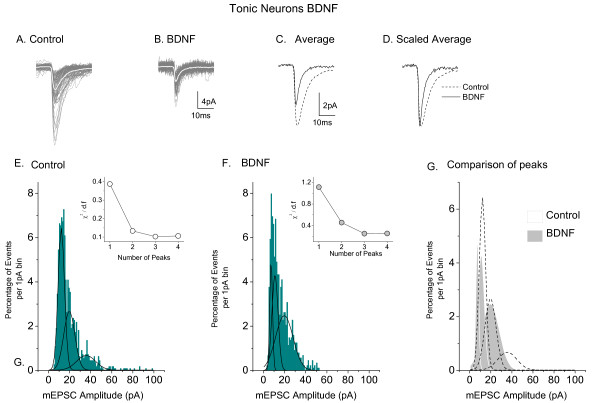
**Analysis of the effects of BDNF on mEPSCs of tonic neurons *(in vitro)***. **A**. Superimposed recordings of 3 min of mEPSC activity in a control tonic neuron, average of events presented as superimposed white trace. **B**. Similar superimposed recordings from a tonic neuron in a BDNF-treated culture. **C**. Averaged events from the neurons illustrated in **A **and **B**. **D**. Averaged events normalized to control size. Note marked increased rate of decay of current. **E**. Distribution histogram (1 pA bins) for amplitudes of 1100 mEPSCs from control tonic neurons. Fit of the data to three Gaussian distributions represented by black lines. **F**. Similar histogram and fit to three Gaussian functions for 877 mEPSCs from BDNF-treated neurons: *Insets *in **E **and **F**. Graphs to show effect of number of Gaussian fits (peaks) on the value of χ^2 ^divided by the number of degrees of freedom. **G**: Superimposition of the three Gaussian peaks obtained in **E **with those obtained in **F**. Modified from reference [24] and reproduced with permission of the Physiological Society.

The observation that BDNF produces a similar 'injury footprint' to CCI (Figure 3F-H) [24,25] raised the possibility that it is alone capable of communication between microglia and neurons in the context of central sensitization. As with CCI, excitatory synaptic drive to delay, irregular, phasic and transient neuron types is increased by BDNF whereas that to tonic neurons is decreased [24,25]. Since many tonic neurons are inhibitory [109,110] and delay neurons are probably never inhibitory [111], we suggested that both BDNF and CCI increase excitatory synaptic drive to excitatory neurons whilst reducing that to inhibitory neurons [24,107].

This similarity was also seen when we used more exacting criteria to identify putative inhibitory cells using both morphological and electrophysiological criteria. Thus both CCI and BDNF reduced excitatory synaptic drive to putative inhibitory tonic islet central neurones (TIC neurons) [25] and increased it to putative excitatory delay radial neurons (DR neurons) [25]. We also identified GABAeric neurons as those which expressed glutamic acid decaboxylase-like immunoreactivity. BDNF also reduced excitatory synaptic drive to these neurons [25].

Although IL-1β increased overall dorsal horn excitability, its effect on tonic and delay neurons differed from that of BDNF and CCI. Thus while IL-1β increased the amplitude of sEPSC's in delay neurons, sEPSC frequency was unaffected and neither the amplitude nor the frequency of sEPSC's were affected in tonic neurons [98]. These observations argue against IL-1β as a major messenger for transfer information between microglia and spinal neurons. It may however be involved in signalling between damaged peripheral tissue and primary afferents [5,6,9,112]

## Further parallels between the actions of BDNF and CCI on tonic neurons

Besides reducing mEPSC and sEPSC amplitude and frequency (Figure 3G), BDNF reduced the time constant for mEPSC decay(τ) in tonic neurons in organotypic culture by 35% [24]. Superimposed events from a typical control tonic neuron and from another neuron from a BDNF-treated culture are shown in Figures 4A and 4B. The white traces show superimposed average data from the two neurons and these are compared in Figure 4C. The scaled averages presented in Figure 4D emphasize the increased rate of mEPSC decay in 'tonic' neurons from BDNF-treated cultures.

**Figure 5:**
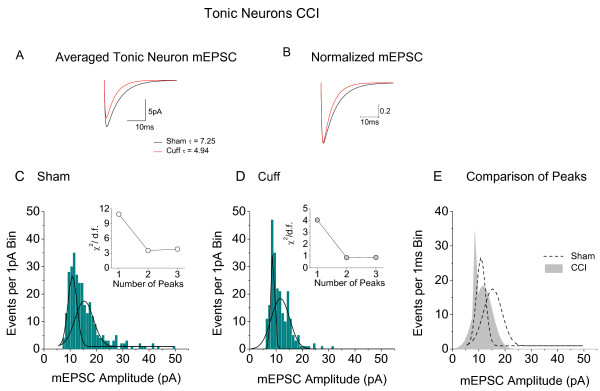
**Analysis of the effects of CCI on mEPSCs of tonic neurons (*ex vivo*)**. **A**. Average mEPSC's in tonic neurons modelled from amplitude and τ values published in [107]. Dark line = average of 325 mEPSC from sham operated animals, red line = average of 175 mEPSC from animals subject to CCI. **B**. Data from A normalized and replotted. **C**. Distribution histogram (1 pA bins) for amplitudes of 325 mEPSCs from tonic neurons in sham operated animals. Fit of the data to two Gaussian distributions represented by black lines. **D**. Similar histogram and fit to two Gaussian functions for 175 mEPSCs in tonic neurons from animals subject to CCI: *Insets *in **C **and **D**. Graphs to show effect of number of Gaussian fits (peaks) on the value of χ^2 ^divided by the number of degrees of freedom. Note that no improvement of fit is seen when a third Gaussian is introduced. **E**: Superimposition of the two Gaussian peaks obtained in **C **with those obtained in **D**.

As well as reducing the amplitude and frequency of mEPSC's and sEPSC's (Figure 3G), CCI produced a 35% reduction in τ in *ex vivo *tonic neurons (see Table 1). Interestingly, this was numerically the same reduction as was seen with BDNF treatment. For sham operated tonic neurons, τ = 7.3 ± 0.3 ms (n = 598) and this was reduced to 4.94 ± 0.62 ms (n = 236) for mEPSCs recorded from animals subject to CCI (t-test, *P < 0.0001*). These numbers as well as the mean mEPSC amplitudes (data from Balasubramanyan et al [107]) were used to model the average events depicted in Figures 5A and 5B; (see Lu et al [24] for methods).

**Table 1:** Comparison of the effects of CCI and BDNF on the characteristics of miniature excitatory postsynaptic currents (mEPSC) in tonic and delay neurons.

		CCI		BDNF		
		*ex vivo *experiments		Organotypic culture experiments		
**Tonic**	τ (mEPSC decay)	↓ 35%		↓ 35%		
		*Peak 1*	*Peak 2*	*Peak 1*	*Peak 2*	*Peak 3*
(sham or control)	mEPSC peak amplitudes	15	10.8	12	19.7	35.7
(CCI or BDNF)	mEPSC peak amplitudes	11.6	8.7	7.3	11	19.4
	Change in mEPSC peak ampltudes	↓23%	↓19%	↓39%	↓44%	↓46%
	Change in **overall **mEPSC amplitude	↓23%		↓18%		
		CCI		BDNF		
**Delay**	τ (mEPSC decay)	Unchanged		↑38%		
		*Peak 1*	*Peak 2*	*Peak 1*	*Peak 2*	*Peak 3*
(sham or control)	mEPSC peak amplitudes	8.5	14.7	9.3	12.7	19
(CCI or BDNF)	mEPSC peak amplitudes	7.6	10.1	8.1	12.5	20.5
	Change in mEPSC peak ampltudes	↓11%	↓31%	↓13%	↓1.5%	↑8%
	Change in **overall **mEPSC amplitude	↑12% Due to appearance of small group of high amplitude responses (Figure 7)		↑10% Due to appearance of small group of high amplitude responses (Figure 6)		

**Figure 6:**
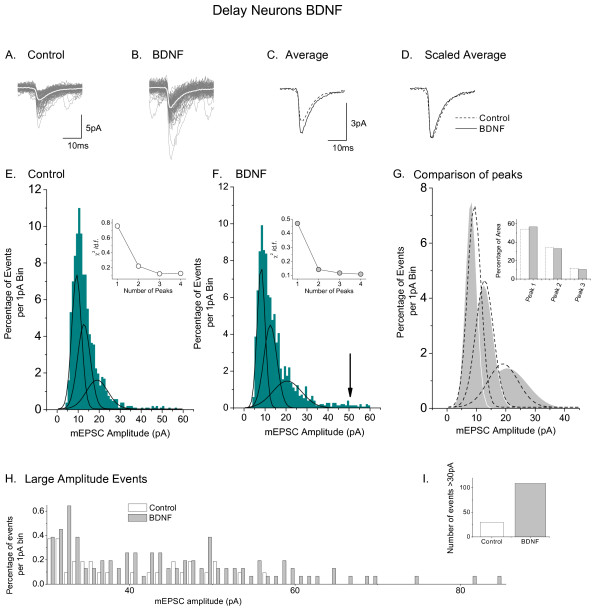
**Analysis of the effects of BDNF on mEPSCs of delay neurons (*in vitro*)**. **A**. Superimposed recordings of 3 min of mEPSC activity in a control delay neuron; average of events presented as superimposed white trace. **B**. Similar superimposed recordings from a delay neuron in a BDNF-treated culture. **C**. Averaged events from the neurons illustrated in **A **and **B**. **D**. Averaged events normalized to control size. Note no change in the rate of decay of current. **E**. Distribution histogram (1 pA bins) for amplitudes of 1074 mEPSCs from control delay neurons. Fit of the data to three Gaussian distributions represented by black lines. **F**. Similar histogram and fit to three Gaussian functions for 1554 mEPSCs from BDNF treated neurons; arrow points out small number of very large events that appear in BDNF. Insets in **E **and **F**, graphs to show effect of number of Gaussian fits (peaks) on the value of *χ*2 divided by the number of degrees of freedom. **G**. Superimposition of the three Gaussian peaks obtained in **E **with those obtained in **F**. Inset, comparison of area under curves for the three peaks. **H**. Data for control and BDNF mEPSCs *>*30 pA replotted and compared on the same axes. **I**. Comparison of number of mEPSC events in control and BDNF treated larger than 30 pA. Modified from reference [24] and used with permission of the Physiological Society.

Three populations of mEPSC amplitudes were identified in control tonic neurons in organotypic slices by fitting Guassian curves to binned histogram data. These appeared at 12.1 ± 0.3, 19.7 ± 2.2 and 35.7 ± 7.4 pA (Figure 4E). By contrast those in BDNF-treated neurons (Figure 4F) had smaller amplitudes with peaks at at 7.3 ± 0.2 and 10.9 ± 1.2 and 19.4 ± 2.4 pA. The insets to Figures 4E and 4F show that fitting with 3 peaks produced the optimal reduction in χ^2 ^(see figure legends for methods). Figure 4G shows superimposed plots of the three Gaussian distributions of mEPSC amplitude from control and BDNF tonic neurons for comparison.

Similar effects were seen in mEPSC population amplitudes in tonic neurons (*ex vivo*) after CCI. Only two populations of mEPSC amplitudes of 15.3 ± 1.3 and 10.8 ± 0.2 pA were seen in tonic neurons from sham operated animals (Figure 5C). Two populations of mEPSC amplitude were also seen in neurons from CCI animals (Figure 5D) but these had smaller peak amplitudes at 11.6 ± 0.1 and 8.7 ± 0.03 pA. The insets to Figures 5C and 5D show that fitting with 2 peaks produced the optimal reduction in χ^2 ^(see Figure legend) with little further reduction in χ^2 ^when a third peak was fitted. Figure 5E shows a superimposition of the Gaussian distributions of mEPSC amplitude from sham and CCI tonic cells for comparison.

Thus for tonic neurons, the effects of CCI and BDNF on both mEPSC time constant of decay (τ) and on the amplitude of subpopulations of mEPSC's are very similar. This similarity is illustrated further in Table 1.

## Further parallels between the actions of BDNF and CCI on delay neurons

Unlike its action on tonic neurons, BDNF did not change the overall τ for recovery of mEPSC in delay neurons in organotypic culture (control τ = 10.7 ± 0.6 ms, n = 766; BDNF τ = 9.5 ± 0.8 ms, n = 1177; t-test, *P > 0.2*). Superimposed individual events from a typical control and a typical BDNF-treated delay neuron are shown in Figures 6A and 6B. Figure 6C shows average data from these cells superimposed. Scaled averages are presented in Figure 6D.

**Figure 7:**
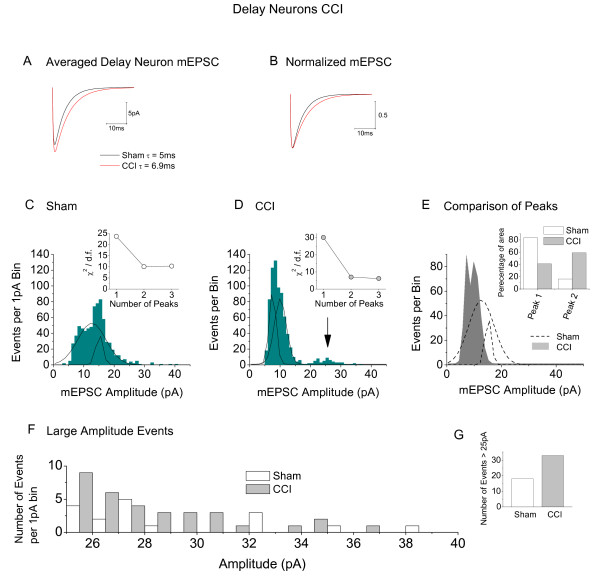
**Analysis of the effects of CCI on mEPSCs of delay neurons (*ex vivo*)**. **A**. Average mEPSC's in delay neurons modelled from amplitude and τ values published in [107]. Dark line = average of 731 mEPSC from sham operated animals, red line = average of 748 mEPSC from animals subject to CCI. **B**. Data from **A **normalized and replotted. **C**. Distribution histogram (1 pA bins) for amplitudes of 731 mEPSCs from delay neurons in sham operated animals. Fit of the data to two Gaussian distributions represented by black lines. **D**. Similar histogram and fit to two Gaussian functions for 748 mEPSCs in tonic neurons from animals subject to CCI: *Insets *in **C **and **D**. Graphs to show effect of number of Gaussian fits (peaks) on the value of χ^2 ^divided by the number of degrees of freedom. Note that no improvement of fit is seen when a third Gaussian is introduced. **E**. Superimposition of the two Gaussian peaks obtained in **C **with those obtained in **D**. Inset, comparison of area under curves for the two peaks. **F**. Data for sham and CCI groups' mEPSCs *>*25 pA replotted and compared on the same axes. **G**. Comparison of number of mEPSC events in sham and CCI delay cells larger than 25 pA.

By contrast, CCI increased τ for mEPSC's in *ex vivo *delay cells. This increased from 5.0 ± 0.1 ms (n = 624) for events from delay neurons in sham animals to 6.9 ± 0.22 ms for events in animals subject to CCI (n = 1066, *P < 0.0001*). These numbers as well as the mean mEPSC amplitudes (data from Balasubramanyan *et al *[107]) were used to model the averaged responses depicted in Figures 7A and 7B; (see Lu *et al *[24] for methods).

Three populations of mEPSC amplitude were identified in control delay neurons in organotypic culture by fitting Gaussian curves to binned histogram data. These appeared at 9.3 ± 1.5, 12.7 ± 9.0 and 19.0 ± 12.3 pA in control neurons (Figure 6E) and at very similar amplitudes (8.1 ± 0.2, 12.5 ± 1.38 and 20.5 ± 4.3 pA) in BDNF-treated delay neurons (Figure 6F). Insets to Figures 6E and 6F show optimized χ^2 ^values for using 3 peaks to fit the data. Figure 6G is a superimposition of the distributions for comparison between control and BDNF-treated neurons. Since BDNF increases overall mEPSC amplitude in delay neurons (Figure 3G), we tested whether changes in the number of events contributing to each of the three distributions could explain this increase. This was done by measuring the area under the Gaussian curves in Figure 6G and expressing the results as percentage of the total area (Figure 6G inset). Surprisingly, similar proportions of the total events made up each of the three peaks under control and BDNF-treated conditions. However, further inspection of the histogram data obtained from BDNF-treated cells revealed a new population of very large events (indicated by arrow in Figure 6F). Whereas only 30 events in the control data had amplitudes >30 pA, 106 events in data from BDNF-treated delay neurons fell into this category. The appearance of this new population of large events is emphasized by the presentation of data for mEPSCs >30 pA in Figures 6H and 6I. Although few events appear in this group, those that do, have large amplitudes. Thus, the emergence of a new group of large mEPSC amplitude events in BDNF may have a noticeable effect on overall mEPSC amplitude.

Only two populations of mEPSC amplitude were seen *ex vivo *in delay neurons from sham operated animals. Peaks appeared at 12.7 ± 0.2 and 15.1 ± 0.1 pA in sham delay cells (Figure 7C) and at (7.6 ± 0.1 and 10.1 ± 0.3 pA) in CCI delay cells (Figure 7D). Insets to Figures 7C and 7D show optimized χ^2 ^values for using 2 peaks to fit the data. Figure 7E is a superimposition of the distributions for comparison between neurons from sham operated animals and those subject to CCI. Whilst the decrease in amplitude of the smaller population is highly significant (*P < 0.0001*), that of the larger population is not (*P > 0.25*). Nevertheless, these data appear to contradict the finding that CCI increases overall mEPSC amplitude in delay neurons (Figure 3F). There are at least two explanations for this discrepancy; first, when we examined the number of events contributing to each of the two peaks (Figure 7E inset) we found that the majority of events in control neurons fell under the smaller peak, whereas after CCI more events contributed to the larger peak. Also, when we examined very large events we found that a small population of very large events appeared in delay neurons from the CCI animals (Figure 7F and 7G). Appearance of this new population of large events is reminiscent of the effect of BDNF on mEPSC's of delay neurons (Figure 6H and 6I). Figure 7G shows that whilst only 18 mEPSC's in neurons from sham operated animals exceeded 25pA, 33 events exceeded this amplitude in neurons from animals subject to CCI.

Table 1 also compares the effects of BDNF and CCI on delay neurons. Although there is some similarity in the consequences of the two manipulations, this is not as obvious as that seen with tonic neurons.

## Conclusions

Many of the findings discussed above are consistent with the possibility that BDNF is alone capable for the transfer of information between activated microglia and neurons during the process of central sensitization. The results supporting this argument are:-

1. In terms of excitatory synaptic transmission, both BDNF and CCI promote a similar 'injury footprint' when the properties of five different neuronal phenotypes are considered (Figure 3) and neither manipulation appears to affect intrinsic neuronal properties such as excitability, input resistance or rheobase [24,107]. Because this injury footprint is not reproduced by IL-1β [98], this argues against its involvement in the final step of transfer of information between microglia to neurons.

2. Detailed analysis of the action of BDNF and CCI on excitatory synaptic transmission to tonic neurons reveals that the two manipulations produce close to identical changes on the kinetics and amplitudes of mEPSC's (Figure 4 and 5, Table 1).

3. A similar analysis of actions on delay neurons show that the actions of CCI and BDNF on mEPSC properties are similar, although not identical (Figure 6 and 7, Table 1).

4. Numerous lines of evidence from Yves De Koninck's laboratory in Quebec and Mike Salter's group in Toronto implicate microglial-derived BDNF in attenuation of Cl^- ^mediated, GABA/glycine inhibition in the dorsal horn [14,86].

5. BDNF and medium from activated microglia both promote an overall increase in dorsal horn excitability (Figure 2) and the effect of the latter is attenuated when BDNF is sequestered using TrkBd5 [25].

If BDNF is sufficient for transferring information between activated microglia and neurons, one has to speculate that other mediators such as IL-1β [20,29,54,113,114], TNF-α[30,96,115], fractalkine [64,65], MCP-1/CCL-2 [18,100,101] and interferon γ [116-118] exert their actions at other points in the cascade of events that initiates central sensitization (Figure 1). The observation that blockade of the action of TNFα with the fusion protein blocker, etanercept, attenuates spinal cord injury induced pain [119], suggest that it may act in series rather than in parallel with BDNF. It may, for example, act in an autocrine fashion to enhance microglial activation [120]. Although our data appear to argue against a role for IL-1β in the microglial-neuron interaction (Figure 3), it is clear that the actions of other mediators need to be studied more carefully. Future experiments will therefore involve an examination of the possible role of TNF-α and interferon-γ in microglia-neuron interactions. Do they mimic the CCI-induced injury footprint in the same way as BDNF? If this is the case, it would still be appropriate to state that BDNF is sufficient to transfer of information between microglia and neurons, but other substances are equally capable of effecting this interaction (i.e. BDNF is 'sufficient' but not 'necessary'). It is also possible that the small discrepancies between the action of BDNF and CCI on excitatory synaptic transmission in delay neurons (Table 1), may reflect actions of mediators other than BDNF. Another issue for future consideration is that the severity, duration and nature of neuronal injury may differentially affect spinal cytokine profile [105]. If this is the case, different mediators may be involved at different points in the sensitization 'cascade' such that some similarity and redundancy of actions of such mediators might be expected.

Lastly, it should be remembered that microglial activation and BDNF release in dorsal horn following injury is transient [66-72], whereas the maintenance of chronic neuropathic pain appears to involve alterations in astrocyte functions [16,17,36-40]. It would therefore be useful to know whether BDNF is involved in astrocytes activation. This interesting possibility remains to be investigated.

## List of Abbreviations

BDNF: Brain derived neurotrophic factor; CCI: Chronic constriction injury (of sciatic nerve); CCL-2/MCP-1: Chemotaxic cytokine ligand 2 (monocyte chemoattractant protein 1); DRG: Dorsal root ganglion; IL-1β: Interleukin 1β; mEPSC: Miniature (TTX resistant) excitatory postsynaptic current; RD neuro: Radial delay neurons; sEPSC: Spontaneous excitatory postsynaptic current; TIC neurons: Tonic islet-central neurons; TNF-α: Tumour necrosis factor-α

## Competing interests

The authors declare that they have no competing interests.

## Authors' contributions

JEB - Review of manuscript and data in Figure 2

VBL - Review of manuscript and data in Figures 4 and 6

SB - Review of manuscript and data in Figures 5 and 7

MJS - Review of manuscript and contribution to data in text on 'RD and TIC neurons'

PAS - Writing first draft of manuscript

All authors have read and approved the final manuscript.
